# Datgan, a reusable software system for facile interrogation and visualization of complex transcription profiling data

**DOI:** 10.1186/1471-2164-12-429

**Published:** 2011-08-24

**Authors:** Gareth R Howell, David O Walton, Benjamin L King, Richard T Libby, Simon WM John

**Affiliations:** 1 The Howard Hughes Medical Institute, Bar Harbor, Maine, USA; 2 The Jackson Laboratory, 600 Main Street, Bar Harbor, Maine, USA; 3 Mount Desert Island Biological Laboratory, Salisbury Cove, ME, USA; 4 University of Rochester Eye Institute, University of Rochester Medical Center, Rochester, NY, USA; 5 Department of Ophthalmology, Tufts University of Medicine, Boston, MA, USA

## Abstract

**Background:**

We introduce Glaucoma Discovery Platform (GDP), an online environment for facile visualization and interrogation of complex transcription profiling datasets for glaucoma. We also report the availability of Datgan, the suite of scripts that was developed to construct GDP. This reusable software system complements existing repositories such as NCBI GEO or EBI ArrayExpress as it allows the construction of searchable databases to maximize understanding of user-selected transcription profiling datasets.

**Description:**

Datgan scripts were used to construct both the underlying data tables and the web interface that form GDP. GDP is populated using data from a mouse model of glaucoma. The data was generated using the DBA/2J strain, a widely used mouse model of glaucoma. The DBA/2J-*Gpnmb^+ ^*strain provided a genetically matched control strain that does not develop glaucoma. We separately assessed both the retina and the optic nerve head, important tissues in glaucoma. We used hierarchical clustering to identify early molecular stages of glaucoma that could not be identified using morphological assessment of disease. GDP has two components. First, an interactive search and retrieve component provides the ability to assess gene(s) of interest in all identified stages of disease in both the retina and optic nerve head. The output is returned in graphical and tabular format with statistically significant differences highlighted for easy visual analysis. Second, a bulk download component allows lists of differentially expressed genes to be retrieved as a series of files compatible with Excel. To facilitate access to additional information available for genes of interest, GDP is linked to selected external resources including Mouse Genome Informatics and Online Medelian Inheritance in Man (OMIM).

**Conclusion:**

Datgan-constructed databases allow user-friendly access to datasets that involve temporally ordered stages of disease or developmental stages. Datgan and GDP are available from http://glaucomadb.jax.org/glaucoma.

## Background

Transcription profiling is a powerful tool for understanding biological process and the role they play in the pathogenesis of disease. For complex diseases, it is necessary to assess many different samples, resulting in very large amounts of data that are cumbersome to analyze and understand. Specific analyses often require significant computing power, time and analytical expertise. These needs hinder detailed interrogation of deposited datasets by many members of the scientific community. Transcription profiling datasets are deposited in central databases such as Gene Expression Omnibus (GEO) at the National Center for Biotechnology Information (NCBI) [1] and ArrayExpress at the European Bioinformatics Institute (EBI) [2]. GEO and ArrayExpress are designed to efficiently store large data sets and to provide mechanisms that allow the scientific community to query, locate, review and download experiments of interest. Although useful, we have found that these platforms are not ideal for optimal interrogation of large and complex datasets. We became aware of the need for a more optimized and facile environment for analyzing complex datasets when studying glaucoma, a complex and asynchronous neurodegenerative disease. Our datasets consists of multiple, temporally ordered stages of disease and existing datasets do not allow simultaneous searching and retrieving of differentially expressed genes in multiple stages of disease compared to a no-disease control.

To meet this need, we developed Glaucoma Discovery Platform (GDP), an online environment to visualize and interrogate large and complex expression datasets in a user-friendly manner. GDP allows simultaneous querying of multiple genes, viewing results across multiple datasets and assessing expression differences for multiple probe sets for each gene. To our knowledge, no other resource is available that provides this combination of user-friendly functionality provided by GDP. We deployed GDP to derive maximum benefit from an extensive transcription profiling study of glaucoma. The study focused on identifying early molecular stages that occur prior to significant damage (described in more detail below and [3]). More than 100 different samples from at least 50 mice were profiled and 70 pairwise group comparisons were made. GDP utilizes a web-based user interface to access the underlying gene expression profiling data, which is organized on a MySQL database server. GDP has greatly facilitated our understanding of these glaucoma data. Its user-friendly interface provides easy and instant data interrogation without a need for specialized knowledge or training. It thereby allows general access to our glaucoma datasets and is freely available for the benefit of the wider scientific community.

We also report the availability of Datgan (Welsh, meaning 'to express'), the suite of scripts that was used to construct both the underlying data tables and the web interface that form GDP. Datgan was written in the python language. It is packaged as a reusable software system for constructing and populating discovery platforms to visualize user-determined sets of transcription profiling data. Datgan is organized to allow biologists with some experience running scripts (or a systems administrator) to establish their own personalized discovery platforms and can be readily adapted to incorporate transcription profiling data generated using RNA-seq.

## Construction and content

### Populating GDP with profiling data for glaucoma

Glaucoma is a complex, neurodegenerative disorder affecting 70 million people worldwide and is associated with the death of retinal ganglion cells (RGCs) and the associated degeneration of the optic nerve [4]. DBA/2J is a widely used mouse model of glaucoma that shows hallmarks of human glaucoma including age-related IOP elevation, optic nerve excavation and regional patterns of RGC loss [5-10]. DBA/2J mice develop glaucoma as a result of a disease of the iris that leads to an elevation in IOP. The disease of the iris is caused by mutations in two genes, *Gpnmb^R150X ^*and *Tyrp1^b^*[5,6]. DBA/2J-*Gpnmb^+ ^*mice have a functioning *Gpnmb *gene and serve as a genetically matched control strain that does not develop glaucoma [11]. An important insult occurs to RGC axons at the optic nerve head in DBA/2J glaucoma [7]. However, other compartments of the RGC also are likely to undergo early changes in glaucoma such as the RGC soma [12] and synapses [13]. The mechanisms involved in these early changes are not well understood.

**Figure 1:**
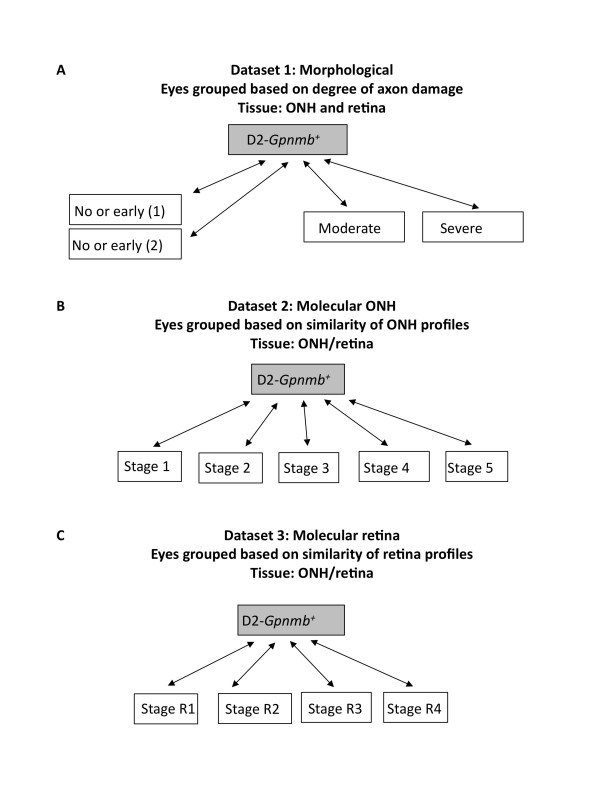
**Datasets available in the first release of GDP**. Each of the three datasets follows a progression through glaucoma. Both optic nerve head and retinal expression data are represented within each dataset [3]. (**a**) Morphological dataset. Glaucoma developing DBA/2J samples (white boxes) and strain-matched D2-*Gpnmb^+ ^*no glaucoma control samples (grey box). Stages of glaucoma were determined morphologically by assessing optic nerve damage just behind the orbit (see Methods and [11,15,34]). (**b**) Molecular ONH dataset. Hierarchical clustering using the expression levels of a set of glaucoma specific genes grouped the optic nerve heads into 5 molecularly defined stages. Stages 1, 2 and 3 represent early states of glaucoma, which precede morphologically detectable glaucoma and so were not previously distinguishable using conventional analyses. Stages 4 and 5 contain eyes with moderate and severe optic nerve damage respectively. (**c**) Molecular retina dataset. A similar hierarchical clustering was performed using the retinal expression data. Four stages of disease were identified with stages R1 and R2 not previously detectable using morphological analysis. Stages R3 and R4 contain eyes with moderate and severe optic nerve damage respectively. Optic nerve heads and retinas were assessed from the same set of eyes.

The gene expression profiling study that is included in GDP, investigated early changes in both the optic nerve head and retina for individual eyes from DBA/2J mice (described in detail elsewhere, [3]). Briefly, the optic nerve head and retina for each eye were separately profiled using Mouse 430 v2 arrays (Affymetrix). 50 DBA/2J eyes and 10 DBA/2J-*Gpnmb^+ ^*control eyes were studied. All data were processed and analyzed using MAANOVA [14]. DBA/2J eyes were initially grouped based on conventional morphological criteria including degree of optic nerve damage (dataset 1: four groups, Figure 1A). However, comparisons of these groups were not sensitive at identifying disease changes that precede morphological damage. Therefore, hierarchical clustering, a method widely used in cancer biology, [7,11,15] was used to group eyes undergoing early stages of disease and allowed much more sensitive detection of early disease changes. Eyes were clustered into different stages using both the expression profiles for the optic nerve head (dataset 2: five stages, Figure 1B) and the retina (dataset 3: four stages, Figure 1C). To identify differentially expressed genes for all three datasets, all possible pairwise comparisons were performed. In total, more than 70 different pairwise comparisons were made and many thousands of differentially expressed genes identified. All raw data has been deposited in NCBI GEO (Accession number: GSE26299).

### Building GDP using Datgan scripts

**Figure 2:**
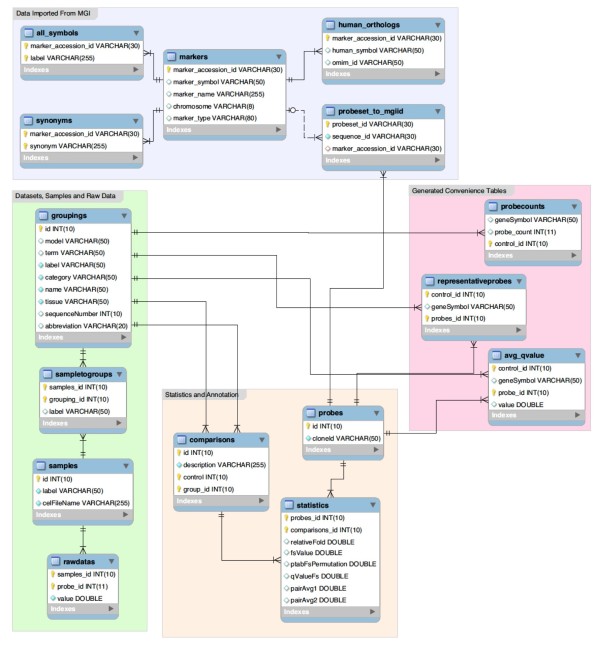
**Schematic of database architecture**. The diagram describes the database schema behind GDP. For convenience, it is organized into four major areas (color coded), (A) Datasets, samples and raw data (green), (B) statistics and annotations (orange), (C) convenience or lookup tables (red) and (D) data imported from MGI (green). Tables are populated in four steps. First, raw normalized intensity values (table name: raw data), samples and probes are added. Second, the analyzed data (statistics, comparisons) and groupings (describing the datasets such as molecular ONH and tissue ONH) are loaded. Third, a previously generated design file allows the associations between samples and groups to be established (sampletogroups). Finally, MGI annotations (all symbols, synonyms, markers, human_orthologs and probeset_to_mgid) are loaded and the convenience look up tables (probecounts, representativeprobes and ave_qvalue) established. Within each table, required columns are indicated. VARCHAR indicates a string is required, and the number in brackets indicates the number of characters allowed in that string. Lines indicated connections between tables.

GDP is constructed as a series of interconnected data tables (Figure 2). Data was loaded in four phases using the first major component of Datgan: First, raw normalized expression values (generated using R/Maanova) were loaded for each probe set for each sample. Second, analyzed data was loaded, including relative fold change and q value, from all pairwise comparisons for all 3 datasets (Morphological, Molecular ONH and Molecular retina). Third, using a previously constructed design file, relationships between the raw data for each sample and the sample groups were established. Finally, gene annotations to the probe sets were loaded from the Mouse Genome Database [16] using public reports available from the Mouse Genome Informatics (MGI) ftp site http://www.informatics.jax.org. This provides the ability not only to search by gene symbols, but also by their synonyms/aliases.

The web interface is implemented using the Ruby on Rails web application framework http://rubyonrails.org in combination with the second major component of Datgan, a series of custom and public Javascript libraries. The interface leverages AJAX technology (asynchronous JavaScript and XML) to allow dynamic regeneration of plots in the same page view, while maintaining the main query panel. The database application infrastructure was implemented in a generic manner, allowing for its reuse for other profiling datasets, and to make it easier to load additional experimental results into GDP. The web-based interactive search and retrieve component provides the ability to assess multiple gene(s) of interest in temporally and/or spatially defined developmental or disease stages. The output is returned in graphical and tabular format with statistically significant differences highlighted for easy visual analysis. Data for all probe sets for a given gene can be accessed as well as all data for individual samples within groups. Additionally, a bulk download component allows lists of differentially expressed genes to be retrieved as a series of tab delimited files. To facilitate access to additional functional information for a given gene, links are provided to external resources.

### Visualizing and interrogating profiling data with GDP

**Figure 3:**
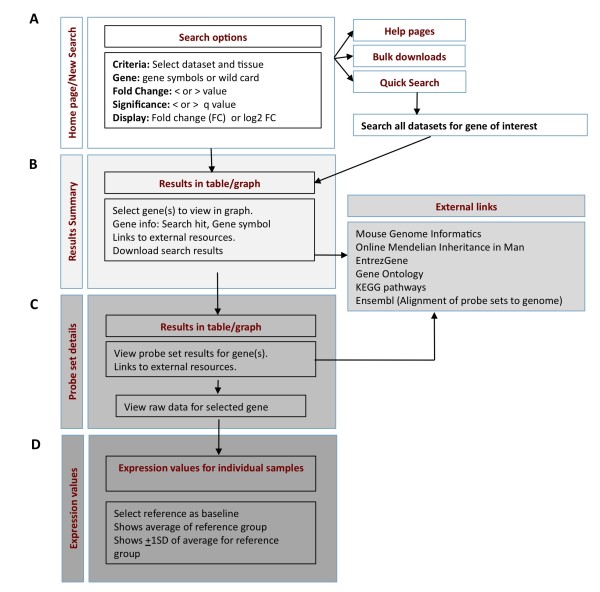
**Schema and Main functions within GDP**. (**a**) The home page contains direct links to users' quick guide, the detailed search tool, bulk downloads and a quick search that interrogates all datasets for a single gene of interest. The detailed search tool enables individual genes, groups of genes or wild card searches (*). The bulk download option enables all differentially expressed genes for pairwise comparisons to be retrieved. (**b**) The gene results page provides both a tabular and graphical view of the expression levels for the searched genes in the different groups for each dataset selected. Access to gene information from external databases is provided (**c**) As multiple probe sets exist for many genes on the Affymetrix 430v2 array, the probe set page details the results for each probe for a chosen gene(s). (**d**) The normalized expression values for individual eyes in each group can be accessed.

The web-based interface is divided into 4 main sections; (a) homepage/new search, (b) results, (c) probe set details and (d) expression values (Figure 3). A number of links are provided to selected external resources such as Mouse Genome Informatics [17], EntrezGene and the Online Medelian Inheritance in Man (OMIM) databases [18] and Kyoto Encyclopedia of Genes and Genomes (KEGG) [19] that allows users to access genetic, phenotypic and functional information for genes of interest (see *External links *below). The platform is also well supported by help pages.

#### Home page/new search (Figure 3a)

The home page provides an overview of the database and permanent headers for convenient links to the bulk download page and the detailed users quick guide. The bulk downloads page allows all differentially expressed genes and associated information for individual comparisons to be downloaded in excel-friendly format. The quick search feature allows a single gene to be searched in all datasets. Information on downloading Datgan is also provided.

**Figure 4:**
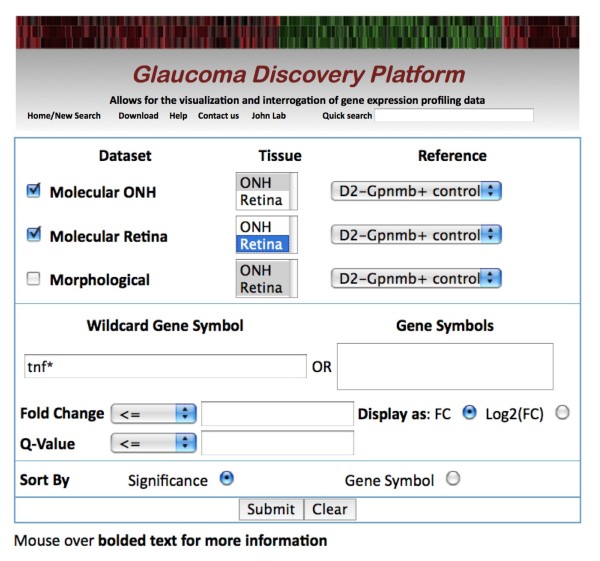
**A detailed search for members of the tumor necrosis factor (TNF) superfamily**. From the search tools box on the home page, 'tnf*' was entered into the 'Wild card Gene Symbol' search box. The search is not case sensitive. Both the 'molecular ONH' dataset (tissue 'ONH') and molecular retina (tissue 'retina') were selected. No limits were set for fold change or q value and the results are to be returned as fold change (FC).

There are a variety of different ways to interrogate the data using the detailed search tool. Firstly, if a user has a set of genes that they are interested in assessing, the official gene symbols (or MGI-recognized aliases) can be entered or pasted into the appropriate text box (Figure 4). Alternatively, there is a wild card (*) capability where, for instance, the results for all members of the tumor necrosis (Tnf) superfamily can be retrieved by searching for "Tnf*" (described in detail below and Figure 4). By default, datasets and tissues "Molecular ONH: tissue ONH" and "Molecular retina: tissue retina" are selected as these are the most sensitive at identifying differentially expressed genes in the optic nerve head and retina respectively. However, the user is also able to select the dataset(s) of interest (see Figure 1), the tissue(s) of interest (optic nerve head or retina) and the reference group (e.g. the D2-*Gpnmb^+ ^*control group). Including additional datasets and tissues does slow the search time. It is possible to restrict the results by fold change and/or significance value (q value). The user can select whether the results are ordered by significance (lowest average q value across all groups) or by gene symbol (alphanumeric). The q value is a measure of the false detection rate and gives an indication of the significance of the fold change. It is a standard statistic for microarray analyses [20]. The lower the q value, the more significant a fold change is considered to be. In our study, genes are considered differentially expressed, with respect to the reference, if the q value is less than 0.05 (roughly equivalent to a false detection rate of 5%). Finally, fold changes can be reported as either relative fold change (compared to reference), or as log_2 _fold change (compared to reference).

#### The results summary page (Figure 3b)

Results are returned in both graphical and tabular format. Any gene names that were not recognized from the search page are listed and links to MGI are given (for clarification of official gene symbols). A 'summary of results' table is shown indicating the number of genes found for each dataset/tissue selected on the search page. Below the summary of results are separate tables and associated graphs for each dataset/tissue searched. Each table contains the gene names searched, the official gene symbol (or description), the representative probe set, and the fold change with associated q value for each stage of glaucoma, compared to the chosen reference group. For ease of identification, significantly differentially expressed values are shown in white on a red background. Genes are ranked in the table based on the chosen option in the detailed search tool (q value or gene symbol). The graph provides a visual display of the fold changes across all stages of disease for the genes of interest, up to a maximum of ten. For searches containing more than 10 genes, the first 10 genes in the table are displayed in the graph. Check boxes are available to the left of each gene to allow the user to select up to 10 genes of choice to view in the graph. Results from the table can be exported as comma separated values (csv, Excel-friendly format). Links to useful publicly available databases are also provided (see *External resources *below).

#### Probe set details page (Figure 3c)

Each gene on the Affymetrix 430 v2 array is interrogated by a probe set of eleven 25 mer probes and a gene can have multiple probe sets [21]. Each probe set corresponds to a particular region of a gene and finding that multiple probes sets for the same gene behave similarly can add confidence to a result. Alternatively, for those probe sets corresponding to alternatively spliced exons/transcripts, multiple probe sets can give insight into the behavior of splice variants [22]. Unfortunately, some probe sets were designed to early versions of gene sequences (prior to accurate genome sequence). This results in some probes in a probe set not being identical to the gene sequence. These probe sets will not accurately reflect the level of transcript for these genes. For each probe set, a link is provided to the Ensembl website where mapping information for each probe set is provided. The values for the representative probe set for each gene is displayed in the results summary page. It is possible to view details for all probe sets for a given gene, to view probe sets for selected genes or for all genes in the table. The probe set detail page provides a table/graph for each gene.

#### Expression values page (Figure 3d)

Values provided in the summary pages are relative fold changes or log_2 _fold changes compared to a selected reference. Each stage of disease contains biological replicates, and the fold change for a given probe set (for a given gene) is calculated as the average of the normalized expression value of all replicates for a defined stage of disease, relative to the reference group. The normalized expression value reflects the relative abundance of the gene in the tissue interrogated (either retina or optic nerve head) with respect to all other genes. Those transcripts with the highest normalized expression value are most abundant. Conversely, those with the lowest normalized expression value are less abundant. An expression level of approximately 4 or less may be considered to represent a gene that is likely not expressed in the assessed tissue (i.e. is close to background levels).

The relative abundance of transcripts corresponding to particular probe sets is important information. For instance, a small fold change in some lowly expressed gene may have greater biological importance than a small fold change in some abundant gene. Knowing the variability in expression levels for different genes in individual eyes within groups also is important. Within any stage of disease (determined either morphologically or molecularly), transcripts within individual biological replicates may behave differently. Genes with low variability within groups may be better targets for intervention strategies than variable genes. The expression values corresponding to each probe set for individual samples (replicates) can be accessed from the probe details page. The expression levels are displayed as a histogram. The average (± 1 standard deviation) for the reference group is indicated.

#### External Resources

Selected external resources can be accessed directly from the results tables (Table 1). Resources were selected to allow users the maximum access to current information for genes of interest. The links to external resources provided by Datgan can be easily adapted for other user-specific databases. A major resource for mouse-based expression is Mouse Genome Informatics (MGI). This resource provides the research community with information on the genetics, genomics and biology of mice [17,23]. EntrezGene and OMIM are databases that form part of the Entrez system at the National Center for Biotechnology Information (NCBI) [18]. EntrezGene provides gene-relevant information such as transcript/protein sequences and links to relevant publications and genome browsers. OMIM provides a simplified disease-oriented description for a given gene including mutations that have been shown to cause diseases in humans. Finally, there are links to The Gene Ontology (GO) database and the Kyoto Encyclopedia of Genes and Genomes (KEGG), two databases that provide functional descriptions of genes. GO provides a controlled vocabulary of terms for biological process, cellular compartments and molecular function and is accessed through MGI [17]. KEGG uses known functional information to construct biologically relevant pathways [19]. Given the uniformity of gene symbols between the external resources and GDP it is possible to identify groups of genes of interest (such as genes in a given KEGG pathway or genes with the same GO term) in the appropriate database and paste these genes into the search tool in GDP.

**Table 1:** Summary of external resources accessed directly from GDP

Database	Acronym	Brief description	Home page address
Mouse Genome Informatics	MGI	Gene catalogues, links to additional resources including phenotype data, genome browsers and SNP databases	http://www.informatics.jax.org/

EntrezGene@NCBI	EntrezGene	Gene catalogues, links to additional resources including genome browsers and SNP databases	http://www.ncbi.nlm.nih.gov/sites/entrez?db=gene

Online Medelian Inheritance in Man	OMIM	Disease-oriented information	http://www.ncbi.nlm.nih.gov/omim/

The Gene Ontology@MGI	GO	Controlled vocabulary for functional attributes	http://www.informatics.jax.org/searches/GO_form.shtml

Kyoto Encyclopedia of Genes and Genomes	KEGG	Biological pathways	http://www.genome.jp/kegg/

Ensembl Genome Browser	Ensembl	Determine alignment of probe sets onto genome sequence	http://www.ensembl.org

### Utility

In this section, we describe the workflow for extracting the data relevant to members of the TNF superfamily. This serves both to reinforce key features of the database described above and as an example that could be followed to interrogate any gene (or group of genes) of interest.

### Assessing members of the tumor necrosis factor (TNF) superfamily

TNF (formerly TNFα) has been suggested to be important in retinal ganglion cell loss during glaucoma [24,25]. Therefore, we assessed all members of the TNF superfamily in both the molecular ONH dataset (Figure 2B, most sensitive dataset of early disease changes in the optic nerve head), and the molecular retina dataset (Figure 2C, most sensitive dataset of early disease changes in the retina).

#### Step 1: Performing detailed search (Figure 4)

First, we determined the best search option. In this case, a wild card search using 'TNF*' will retrieve all TNF superfamily members as no *a priori *knowledge about which genes are present in the TNF superfamily is required. Second, we selected the datasets and tissues of interest - molecular ONH dataset, tissue 'ONH' and molecular retina dataset, tissue 'retina'. Finally, we selected the reference group; in this case, D2-*Gpnmb^+ ^*control eyes. In this example, we did not limit our search based on fold change or q value, and chose the default options for the layout of the results page (display expression differences as fold change rather than log_2 _of fold change, and ordered genes based on significance of gene expression differences (q value) rather than gene symbol).

#### Step 2: Visualizing the results (Figure 5)

**Figure 5:**
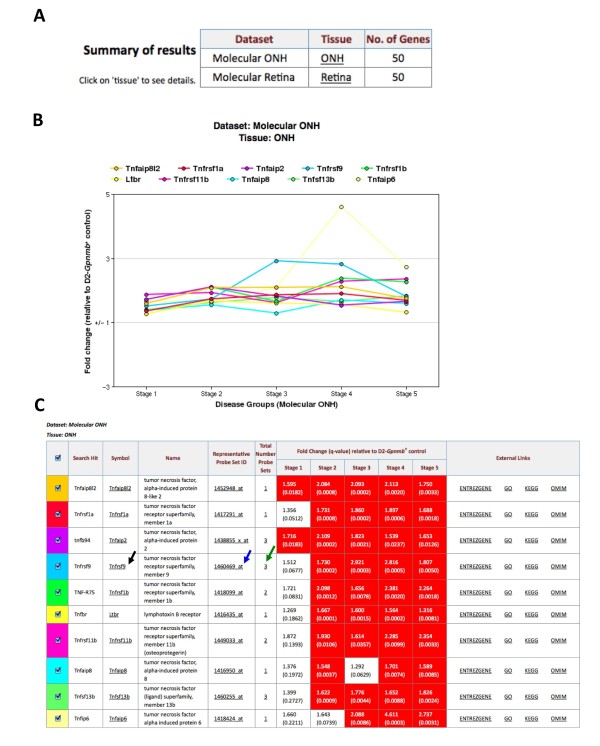
Results of the wild card search TNF*. **(a) **The summary of the results indicates the number of genes identified in each of the tissues and datasets searched. (**b-c**) Result details are returned in graphical (b) and tabular format (c). For the molecular ONH dataset, the relative fold change (with respect to the chosen reference) and the q value for the representative probe set for each gene is provided. The representative probe set for each gene is determined as the probe set with the lowest average q value across all groups. The ten most significant differentially expressed genes in the TNF superfamily are shown. Links are provided for each gene to selected external resources. These are MGI - by clicking on the gene symbol (black arrow), Ensembl (by clicking the representative probe ID, blue arrow) EntrezGene, OMIM, GO and KEGG (database (DB) links, far left). Details of all probe sets for a given gene can be accessed (green arrow).

The results were returned below the original search box, allowing straightforward modification of search options if necessary. First, the summary table indicates that 50 genes met the criteria of the search for both the molecular ONH and molecular retina datasets (Figure 5A). These 50 genes have 'TNF' in either their official gene symbol or in any MGI-approved alias(es). Below the summary of results table, detailed results for each dataset are displayed. In this example, we assess the results for the molecular ONH dataset, tissue ONH. First, the top 10 most significant genes are visualized in the graph (Figure 5B). The results for all 50 genes are shown in the table, the top 10 most significant of which are shown in Figure 5C. Of the 50 genes, 34 are differentially expressed in at least one stage in the molecular ONH dataset. To download all expression data in the table, use the 'Download as comma separated values (CSV)' option below the table. Expression values (and associated q values) for the 50 genes across all stages of disease are exported in excel-friendly format (Table 2).

**Table 2:** All 'Tnf'-related genes in the molecular optic nerve head dataset

Searched	Symbol	Representative Probe Set ID	Num Probe Sets	FC for Stage 1	QV for Stage 1	FC for Stage 2	QV for Stage 2	FC for Stage 3	QV for Stage 3	FC for Stage 4	QV for Stage 4	FC for Stage 5	QV for Stage 5
*Tnfaip8l2*	*Tnfaip8l2*	1452948_at	1	1.595	0.0182	2.084	0.0008	2.093	0.0002	2.113	0.002	1.75	0.0033
*TNFR60*	*Tnfrsf1a*	1417291_at	1	1.356	0.0512	1.731	0.0008	1.86	0.0002	1.897	0.0006	1.688	0.0018
*tnfb94*	*Tnfaip2*	1438855_x_at	3	1.716	0.0183	2.109	0.0002	1.823	0.0021	1.539	0.0237	1.653	0.0126
*Tnfrsf9*	*Tnfrsf9*	1460469_at	3	1.512	0.0677	1.73	0.0002	2.921	0.0003	2.816	0.0005	1.807	0.005
*TNF-alphaR2*	*Tnfrsf1b*	1418099_at	2	1.721	0.0831	2.098	0.0012	1.656	0.0078	2.381	0.002	2.264	0.0018
*TNFCR*	*Ltbr*	1416435_at	1	1.269	0.1862	1.667	0.0001	1.6	0.0015	1.564	0.0002	1.316	0.0081
*Tnfrsf11b*	*Tnfrsf11b*	1449033_at	2	1.872	0.1393	1.93	0.0106	1.614	0.0357	2.285	0.0099	2.354	0.0033
*Tnfaip8*	*Tnfaip8*	1416950_at	1	1.376	0.1972	1.548	0.0037	1.292	0.0629	1.701	0.0074	1.589	0.0085
*Tnfsf13b*	*Tnfsf13b*	1460255_at	3	1.399	0.2727	1.622	0.0009	1.776	0.0044	1.652	0.0088	1.826	0.0024
*Tnfaip6*	*Tnfaip6*	1418424_at	1	1.66	0.2211	1.643	0.0739	2.088	0.0086	4.611	0.0003	2.737	0.0031
*Tnfrsf13b*	*Tnfrsf13b*	1423182_at	1	1.284	0.272	1.79	0.0003	1.34	0.0408	2.029	0.0007	1.679	0.0066
*Tnfip1*	*Tnfaip1*	1448863_a_at	3	1.113	0.3315	1.295	0.0027	1.297	0.0015	1.469	0.0006	1.263	0.0025
*Tnfrsf22*	*Tnfrsf22*	1426095_a_at	4	1.331	0.3691	1.681	0.0098	1.672	0.0141	1.95	0.0048	1.537	0.009
*Tnfrsf6*	*Fas*	1460251_at	5	1.228	0.4172	1.545	0.0041	1.831	0.0038	1.755	0.0048	1.982	0.0025
*Tnfrsf21*	*Tnfrsf21*	1450731_s_at	2	1.166	0.3452	1.251	0.0098	1.363	0.0039	1.275	0.0225	1.218	0.0779
*Tnfrsf12a*	*Tnfrsf12a*	1418571_at	2	1.301	0.3733	1.497	0.0759	2.851	0.0003	3.947	0.0002	1.941	0.0102
*Tnfaip9*	*Steap4*	1460197_a_at	2	1.177	0.5803	1.734	0.0044	1.576	0.0295	1.563	0.0477	1.832	0.0061
*Tnfrsf5*	*Cd40*	1439221_s_at	3	1.144	0.4776	1.238	0.0049	1.438	0.0067	1.72	0.0057	1.122	0.183
*Tnfrsf10b*	*Tnfrsf10b*	1421296_at	2	1.335	0.3902	1.694	0.0074	1.609	0.0227	1.651	0.0194	1.099	0.3627
*Tnfsf13*	*Tnfsf13*	1418345_at	1	1.077	0.721	1.357	0.0094	1.328	0.0281	1.303	0.0875	1.431	0.0147
*Tnfip3*	*Tnfaip3*	1433699_at	2	-1.182	0.5997	-1.476	0.0076	-1.295	0.1286	-1.459	0.0288	-1.187	0.1717
*Tnfrsf11a*	*Tnfrsf11a*	1430259_at	2	1.105	0.5704	1.279	0.022	1.092	0.2354	1.252	0.1037	1.207	0.1559
*Tnfa*	*Tnf*	1419607_at	1	1.068	0.6746	1.164	0.1341	1.173	0.2162	1.197	0.0413	1.157	0.088
*Tnfsf10*	*Tnfsf10*	1459913_at	3	1.231	0.432	1.399	0.014	1.102	0.4007	1.134	0.3464	1.809	0.0027
*Tnfrsf19*	*Tnfrsf19*	1448147_at	3	1.281	0.2536	1.536	0.0052	1.023	0.5083	1.176	0.3387	1.208	0.0931
*Tnfrsf13c*	*Tnfrsf13c*	1419307_at	1	1.084	0.7361	1.172	0.2768	-1.554	0.0445	-1.61	0.0562	-1.395	0.0958
*Tnfrsf18*	*Tnfrsf18*	1422303_a_at	1	1.113	0.5149	1.268	0.053	1.24	0.0632	1.114	0.2903	1.072	0.2896
*Tnfsf3l*	*Pglyrp1*	1449184_at	1	-1.022	0.7984	1.241	0.1306	1.401	0.0694	1.273	0.1955	-1.363	0.099
*TNF-beta*	*Lta*	1420353_at	1	1.165	0.3119	1.054	0.3871	1.059	0.3635	1.118	0.2847	1.194	0.0614
*Tnfrsf14*	*Tnfrsf14*	1452425_at	1	1.034	0.7298	1.181	0.0819	1.08	0.2514	1.075	0.3767	1.196	0.0106
*Tnfsf14*	*Tnfsf14*	1450298_at	2	1.11	0.4399	1.213	0.0478	1.05	0.4048	1.192	0.161	1.037	0.4038
*Tnfsf8*	*Tnfsf8*	1450272_at	2	1.049	0.682	1.146	0.1413	1.105	0.2307	1.128	0.1819	1.076	0.3008
*Tnfsf7*	*Cd70*	1449926_at	1	1.075	0.6845	1.202	0.1024	1.09	0.3569	1.54	0.023	-1.028	0.439
*Tnfsf11*	*Tnfsf11*	1419083_at	2	1.121	0.65	1.3	0.1171	1.144	0.2947	1.013	0.5497	1.517	0.0236
*Tnfrsf16*	*Ngfr*	1454903_at	3	-1.085	0.6105	1.019	0.5138	1.023	0.5083	-1.606	0.0053	-1.449	0.0062
*Tnfsf6*	*Fasl*	1449235_at	2	1.037	0.7033	1.133	0.1216	1.115	0.2646	1.016	0.5184	1.253	0.05
*Tnfrsf7*	*Cd27*	1452389_at	1	1.05	0.6727	1.168	0.1289	1.177	0.165	1.098	0.2723	-1.001	0.4951
*Tnfrsf25*	*Tnfrsf25*	1422231_a_at	1	1.081	0.5818	1.112	0.2304	1.071	0.3705	1.086	0.353	-1.03	0.434
*Tnfsf4*	*Tnfsf4*	1421744_at	1	1.014	0.7928	1.112	0.2484	1.055	0.3972	1.045	0.4892	1.284	0.0442
*Tnfsf5*	*Cd40lg*	1422283_at	1	-1.032	0.7476	-1.093	0.1604	-1	0.546	-1.014	0.5263	1.187	0.0304
*Tnfsf9*	*Tnfsf9*	1422924_at	1	1.036	0.7722	1.119	0.3593	1.145	0.3207	-1.027	0.5346	1.363	0.0246
*Tnfrsf23*	*Tnfrsf23*	1422101_at	1	-1.003	0.814	1.024	0.5169	1.035	0.4692	1.268	0.1378	1.215	0.109
*Tnfaip8l1*	*Tnfaip8l1*	1449125_at	1	1.039	0.7492	1.073	0.3754	1.114	0.231	1.118	0.3217	1.039	0.3833
*Tnfsf3*	*Ltb*	1419135_at	1	-1.076	0.7049	1.057	0.3884	1.026	0.4906	1.142	0.2959	-1.122	0.2268
*Tnfrsf19l*	*Relt*	1455116_at	1	-1.055	0.7184	-1.009	0.5445	-1.151	0.1717	-1.124	0.3145	-1.043	0.4081
*Tnfsf12*	*Tnfsf12*	1452440_at	1	1.03	0.7513	-1.05	0.4461	1.066	0.3841	1.094	0.3333	1.107	0.2445
*Tnfsf5ip1*	*Psmg2*	1425373_a_at	2	-1.067	0.6716	-1.082	0.3645	1.119	0.1569	1.013	0.5411	-1.017	0.4574
*Tnfrsf13*	*Tnfrsf17*	1420782_at	1	-1.072	0.6676	-1.051	0.4316	-1.087	0.37	-1.033	0.5123	1.062	0.3875
*Tnfrsf4*	*Tnfrsf4*	1420351_at	1	-1.041	0.7555	-1.019	0.5272	1.047	0.4614	1.022	0.5351	1.031	0.4478
*Tnfrsf8*	*Tnfrsf8*	1421649_at	1	-1.002	0.8147	-1.005	0.5523	-1.028	0.4824	-1.001	0.5686	-1.051	0.3787

FC = Fold change, QV = Q value

#### Step 3: Interpreting the results (Figure 6)

*Tnf *shows only a modest increase in expression in stage 4 (1.2 fold, q value = 0.0413) in the molecular ONH dataset. The five most significant genes are Tnf, alpha-induced protein 8-like 2 (*Tnfaip8l2*), Tnf superfamily receptor 1a (*Tnfrsf1a*, formerly *Tnfr1*) and 1b (*Tnfrsf1b*, formerly *Tnfr2*), Tnf alpha-induced protein 2 (*Tnfaip2*) and Tnf superfamily member 9 (*Tnfrsf9*) (Figure 5C, Table 2). *Tnfrsf9 *has the highest fold change of all TNF-related genes in the molecular ONH dataset, with a 2.9 fold expression difference compared to D2-*Gpnmb^+ ^*controls in stage 3. Of direct relevance to DBA/2J glaucoma, *Tnfrsf9 *is involved in the proliferation of monocytes that are precursors of microglia and macrophages [26-28]. This information was retrieved from either OMIM or Entrez Gene. Microglia/macrophages have been shown to increase in the optic nerve head and retina early in glaucoma [3,29].

**Figure 6:**
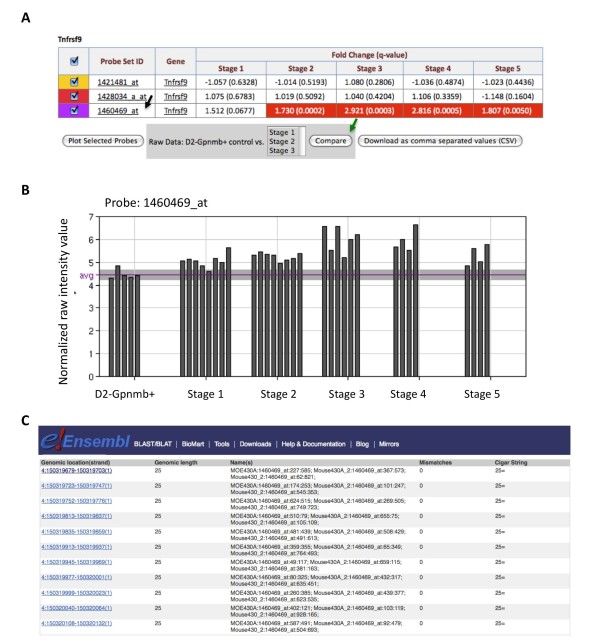
**Analyzing multiple probe sets for *Tnfrsf9 *suggests that only the major transcript is expressed in the optic nerve head**. **(a) **The table from the probe set details page for the *Tnfrsf9 *gene (a graphical view is also available - not shown). Three probe sets are available for this gene. Only one probe set, 1460469_at, is differentially expressed in the optic nerve head. The remaining two probes show no difference with respect to D2-*Gpnmb^+ ^*control group. (**b**) The normalized expression values (intensity values) for the 1460469_at probe set for the molecular ONH stages. This graph shows the variability of *Tnfrsf9 *expression in individual eyes. The normalized raw intensity values range from between 4 and 7. The average of the reference group is shown (purple line) along with one standard deviation above and below the average (grayed area). (**c**) Detailed mapping data for all probes within a probe set can be accessed in Ensembl by clicking on the probe set name in the results table (black arrow, a). For each probe set, the genomic location of each of the eleven 25 mer probes that make up a probe set and any mismatches are reported. For 146049_at, all probes match genomic intervals within the *Tnfrsf9 *gene. This is not the case for 1421481_at or 1428034_a_at (data not shown).

As described above, many genes are represented by multiple probe sets on the Affymetrix 430 v2 array. *Tnfrsf9 *is represented on the array by three probe sets (1460469_at, 1428034_a_at and 1421481_at) with '1460469_at' being shown in the results table as the most significant probe set of the three. Given the importance of all probe sets, full information can be accessed through the probe set details page (Figure 3C and 6). Only 1460469_at is differentially expressed in the molecular ONH dataset, tissue ONH, the other two probe sets show no significant difference compared to the D2-*Gpnmb^+ ^*control group (Figure 6A). To view the normalized expression levels of the probe sets in the optic nerve head it is necessary to access the expression values page (Figure 6B). The normalized raw intensity values for individual eyes for the DE probe set 1460469_at range between 4 (considered background) and 7. The raw normalized values for the other non-DE probe sets do not increase significantly above background.

The differing expression levels for the three *Tnfrsf9 *probe sets may result from detection of alternative splice forms by the different probe sets or from errors in the original probe set designs. The apparent differences in expression levels between *Tnfrsf9 *probe sets are likely due to probe sequence errors. Detailed data for all probes within each probe set is available in Ensembl and can be accessed directly from GDP. Only for probe set 1460469_at do all eleven probes match identically within the *Tnfrsf9 *gene (Figure 6c). Ensembl does not report an alignment for any probes in the 1421481_at probe set. For the remaining probe set, 1428034_a_at, three of the eleven probes have mismatches, and another three probes include intronic sequences that would not be included in the messenger RNA. This explains a lack of expression for this probe set as an average of the expression level of all probes is taken. Given this detailed probe information, it is clear that the expression level of *Tnfrsf9 *is only truly represented by the 1460469_at probe set.

In summary, using GDP, we have readily performed a detailed search of all TNF superfamily members and readily identified all genes that change significantly compared to controls in two out of three different datasets. In particular, the expression of *Tnfrsf9 *increases in the optic nerve head at very early stages of glaucoma and may be related to an accumulation of microglia. These results are of great interest to the glaucoma community and are immediately and freely available. They highlight major advantages of accessing these gene expression datasets through GDP, and the utility of Datgan for providing similar environments for many other datasets.

## Discussion

Here, we describe GDP, constructed using the reusable software system Datgan, for visualizing and interrogating complex gene expression profiling data. Datgan is freely available for download (via http://www.simonjohnlab.org) and can be used to construct platforms for viewing and interrogating any set of microarray or RNA-seq based transcription profiling datasets. Datgan will be particularly useful for complex diseases that have variable onset and progression. As exemplified by GDP, platforms developed using Datgan allow general access and improved understanding of complex datasets.

Use of GDP is proving very important for understanding our glaucoma datasets. For publication in most journals, all profiling datasets are required to be deposited in either GEO (NCBI) or ArrayExpress (EBI). All data and associated files for our study were deposited in NCBI GEO (Accession number GSE26299). Data deposition in these archives is necessary to ensure access to all the raw data for interested parties. However, these archives were not designed to allow detailed interrogation of multiple genes or biological pathways across multiple datasets. GDP includes this functionality and massively facilitates interrogation of our glaucoma datasets by us, and the scientific community, ultimately allowing the most benefit to be derived from the data. Other gene expression profiling studies relevant to glaucoma, which have been or will be carried out, also could be incorporated into GDP. Many investigators, irrespective of computational expertise, can perform detailed 'on the fly' searches quickly and easily.

Datgan is specifically designed to allow additional datasets to be easily incorporated after the initial online environment has been constructed. For glaucoma, other gene expression profiling studies have been carried out that could be included into GDP. These include studies that have profiled additional animal models of glaucoma [30-32]. In addition, comparing cell- and tissue- specific transcription profiling studies (e.g [33]) in non-diseased settings would enable hypotheses to be made about which cells are changing early in glaucoma. Finally, useful predictions could be made by comparing studies that have profiled other relevant diseases such as other neurodegenerative disorders or diseases affecting retinal ganglion cells.

## Conclusion

Datgan is a powerful software package for developing user-friendly platforms to visualize transcription profiling data. Implemented as GDP, it is already proving an essential tool for interrogating the molecular pathogenesis of glaucoma.

## Availability and Requirements

The database can be accessed via http://glaucomadb.jax.org/glaucoma. Web browsers: Tested on the major web browsers including the latest versions of Firefox, Safari and Chrome. It has also been tested on smart phones and tablet computers. Operating system(s): Tested on SUSE Linux Enterprise Server 11 & OpenSUSE 11.0, and should work on other versions of Linux and Mac OSX. Tools required to establish Datgan-derived databases include: Ruby 1.8.7, Rails 2.3.5, Python 2.6, Percona-Server-5.5.13 (should work with most MySQL), apache2, Mongrel Web Server 1.1.5. Source code details can be obtained from the Datgan tab at the top of the home page.

## Authors' contributions

GRH, RTL and SWMJ designed the database and contributed to manuscript preparation. BLK contributed to the design and implemented an initial prototype version of the database. DOW wrote the Datgan suites, constructed and populated Glaucoma Discovery Platform. All authors read and approved the final manuscript.

## References

[B1] Barrett T Troup DB Wilhite SE Ledoux P Rudnev D Evangelista C Kim IF Soboleva A Tomashevsky M Edgar R NCBI GEO: mining tens of millions of expression profiles--database and tools update Nucleic Acids Res 2007 35 Database D760 765 10.1093/nar/gkl887 17099226 PMC1669752

[B2] Parkinson H Kapushesky M Shojatalab M Abeygunawardena N Coulson R Farne A Holloway E Kolesnykov N Lilja P Lukk M ArrayExpress--a public database of microarray experiments and gene expression profiles Nucleic Acids Res 2007 35 Database D747 750 10.1093/nar/gkl995 17132828 PMC1716725

[B3] Howell GR Macalinao DG Sousa GS Walden M Soto I Kneeland SL Barbay JM King BL Marchant JK Hibbs M Molecular clustering identifies complement and endothelin induction as early events in a mouse model of glaucoma J.Clinical Investigation 121 4 1429 1444 10.1172/JCI44646 PMC3069778 21383504

[B4] Quigley HA Broman AT The number of people with glaucoma worldwide in 2010 and 2020 Br J Ophthalmol 2006 90 3 262 267 10.1136/bjo.2005.081224 16488940 PMC1856963

[B5] Anderson MG Smith RS Hawes NL Zabaleta A Chang B Wiggs JL John SW Mutations in genes encoding melanosomal proteins cause pigmentary glaucoma in DBA/2J mice Nat Genet 2002 30 1 81 85 10.1038/ng794 11743578

[B6] Chang B Smith RS Hawes NL Anderson MG Zabaleta A Savinova O Roderick TH Heckenlively JR Davisson MT John SW Interacting loci cause severe iris atrophy and glaucoma in DBA/2J mice Nat Genet 1999 21 4 405 409 10.1038/7741 10192392

[B7] Howell GR Libby RT Jakobs TC Smith RS Phalan FC Barter JW Barbay JM Marchant JK Mahesh N Porciatti V Axons of retinal ganglion cells are insulted in the optic nerve early in DBA/2J glaucoma J Cell Biol 2007 179 7 1523 1537 10.1083/jcb.200706181 18158332 PMC2373494

[B8] Jakobs TC Libby RT Ben Y John SW Masland RH Retinal ganglion cell degeneration is topological but not cell type specific in DBA/2J mice J Cell Biol 2005 171 2 313 325 10.1083/jcb.200506099 16247030 PMC2171185

[B9] Libby RT Anderson MG Pang IH Robinson ZH Savinova OV Cosma IM Snow A Wilson LA Smith RS Clark AF Inherited glaucoma in DBA/2J mice: pertinent disease features for studying the neurodegeneration Vis Neurosci 2005 22 5 637 648 16332275 10.1017/S0952523805225130

[B10] Schlamp CL Li Y Dietz JA Janssen KT Nickells RW Progressive ganglion cell loss and optic nerve degeneration in DBA/2J mice is variable and asymmetric BMC Neurosci 2006 7 66 10.1186/1471-2202-7-66 17018142 PMC1621073

[B11] Howell GR Libby RT Marchant JK Wilson LA Cosma IM Smith RS Anderson MG John SW Absence of glaucoma in DBA/2J mice homozygous for wild-type versions of *Gpnmb *and *Tyrp1* BMC Genet 2007 8 1 45 17608931 10.1186/1471-2156-8-45 PMC1937007

[B12] Soto I Oglesby E Buckingham BP Son JL Roberson ED Steele MR Inman DM Vetter ML Horner PJ Marsh-Armstrong N Retinal ganglion cells downregulate gene expression and lose their axons within the optic nerve head in a mouse glaucoma model J Neurosci 2008 28 2 548 561 10.1523/JNEUROSCI.3714-07.2008 18184797 PMC6670511

[B13] Stevens B Allen NJ Vazquez LE Howell GR Christopherson KS Nouri N Micheva KD Mehalow AK Huberman AD Stafford B The classical complement cascade mediates CNS synapse elimination Cell 2007 131 6 1164 1178 10.1016/j.cell.2007.10.036 18083105

[B14] Cui X Churchill GA Statistical tests for differential expression in cDNA microarray experiments Genome Biol 2003 4 4 210 10.1186/gb-2003-4-4-210 12702200 PMC154570

[B15] Libby RT Li Y Savinova OV Barter J Smith RS Nickells RW John SW Susceptibility to neurodegeneration in a glaucoma is modified by Bax gene dosage PLoS Genet 2005 1 1 17 26 10.1371/journal.pgen.0010017 16103918 PMC1183523

[B16] Bult CJ Eppig JT Kadin JA Richardson JE Blake JA The Mouse Genome Database (MGD): mouse biology and model systems Nucleic Acids Res 2008 36 Database D724 728 18158299 10.1093/nar/gkm961 PMC2238849

[B17] Blake JA Bult CJ Eppig JT Kadin JA Richardson JE The Mouse Genome Database genotypes::phenotypes Nucleic Acids Res 2009 37 Database D712 719 10.1093/nar/gkn886 18981050 PMC2686566

[B18] Sayers EW Barrett T Benson DA Bryant SH Canese K Chetvernin V Church DM DiCuccio M Edgar R Federhen S Database resources of the National Center for Biotechnology Information Nucleic Acids Res 2009 37 Database D5 15 10.1093/nar/gkn741 18940862 PMC2686545

[B19] Aoki KF Kanehisa M Using the KEGG database resource Curr Protoc Bioinformatics 2005 Chapter 1 Unit 1 12 10.1002/0471250953.bi0112s11 18428742

[B20] Churchill GA Using ANOVA to analyze microarray data Biotechniques 2004 37 2 173 175 177 15335204 10.2144/04372TE01

[B21] Irizarry RA Bolstad BM Collin F Cope LM Hobbs B Speed TP Summaries of Affymetrix GeneChip probe level data Nucleic Acids Res 2003 31 4 e15 10.1093/nar/gng015 12582260 PMC150247

[B22] Stalteri MA Harrison AP Interpretation of multiple probe sets mapping to the same gene in Affymetrix GeneChips BMC Bioinformatics 2007 8 13 10.1186/1471-2105-8-13 17224057 PMC1784106

[B23] Shaw DR Searching the Mouse Genome Informatics (MGI) resources for information on mouse biology from genotype to phenotype Curr Protoc Bioinformatics 2009 Chapter 1 Unit1 7 10.1002/0471250953.bi0107s25 19274630

[B24] Nakazawa T Nakazawa C Matsubara A Noda K Hisatomi T She H Michaud N Hafezi-Moghadam A Miller JW Benowitz LI Tumor necrosis factor-alpha mediates oligodendrocyte death and delayed retinal ganglion cell loss in a mouse model of glaucoma J Neurosci 2006 26 49 12633 12641 10.1523/JNEUROSCI.2801-06.2006 17151265 PMC6674838

[B25] Tezel G Li LY Patil RV Wax MB TNF-alpha and TNF-alpha receptor-1 in the retina of normal and glaucomatous eyes Invest Ophthalmol Vis Sci 2001 42 8 1787 1794 11431443

[B26] Jiang D Chen Y Schwarz H CD137 induces proliferation of murine hematopoietic progenitor cells and differentiation to macrophages J Immunol 2008 181 6 3923 3932 18768847 10.4049/jimmunol.181.6.3923

[B27] Langstein J Michel J Fritsche J Kreutz M Andreesen R Schwarz H CD137 (ILA/4-1BB), a member of the TNF receptor family, induces monocyte activation via bidirectional signaling J Immunol 1998 160 5 2488 2494 9498794

[B28] Langstein J Michel J Schwarz H CD137 induces proliferation and endomitosis in monocytes Blood 1999 94 9 3161 3168 10556203

[B29] Bosco A Steele MR Vetter ML Early microglia activation in a mouse model of chronic glaucoma J Comp Neurol 519 4 599 620 10.1002/cne.22516 PMC4169989 21246546

[B30] Steele MR Inman DM Calkins DJ Horner PJ Vetter ML Microarray analysis of retinal gene expression in the DBA/2J model of glaucoma Invest Ophthalmol Vis Sci 2006 47 3 977 985 10.1167/iovs.05-0865 16505032

[B31] Johnson EC Jia L Cepurna WO Doser TA Morrison JC Global changes in optic nerve head gene expression after exposure to elevated intraocular pressure in a rat glaucoma model Invest Ophthalmol Vis Sci 2007 48 7 3161 3177 10.1167/iovs.06-1282 17591886 PMC1950563

[B32] Yang Z Quigley HA Pease ME Yang Y Qian J Valenta D Zack DJ Changes in Gene Expression in Experimental Glaucoma and Optic Nerve Transection: The Equilibrium between Protective and Detrimental Mechanisms Invest Ophthalmol Vis Sci 2007 48 12 5539 5548 10.1167/iovs.07-0542 18055803

[B33] Cahoy JD Emery B Kaushal A Foo LC Zamanian JL Christopherson KS Xing Y Lubischer JL Krieg PA Krupenko SA A transcriptome database for astrocytes, neurons, and oligodendrocytes: a new resource for understanding brain development and function J Neurosci 2008 28 1 264 278 10.1523/JNEUROSCI.4178-07.2008 18171944 PMC6671143

[B34] Anderson MG Libby RT Gould DB Smith RS John SW High-dose radiation with bone marrow transfer prevents neurodegeneration in an inherited glaucoma Proc Natl Acad Sci USA 2005 102 12 4566 4571 10.1073/pnas.0407357102 15758074 PMC555465

